# Global Research Trends in Physical Activity Barriers: A Study on Men

**DOI:** 10.3390/healthcare12202098

**Published:** 2024-10-21

**Authors:** Huseyin Gumus, Mustafa Can Koc, Laurentiu-Gabriel Talaghir

**Affiliations:** 1 Faculty of Sports Sciences, Mersin University, 33110 Mersin, Türkiye; huseyinn.gumuss@gmail.com; 2 Faculty of Sports Sciences, Istanbul Gelisim University, 34310 Istanbul, Türkiye; 3 Directorate of Sports Sciences Application and Research Center, Istanbul Gelisim University, 34310 Istanbul, Türkiye; 4 Faculty of Physical Education and Sport, Dunarea de Jos University of Galati, 800008 Galati, Romania

**Keywords:** men, physical activity, barriers, bibliometric analysis, systematic review, research trends

## Abstract

In this study, bibliometric properties of studies on physical activity barriers in men were analyzed using the Biblioshiny interface in R programming language. According to the results of the analysis, a total of 867 articles were published in 397 journals between 1992 and 2024. A great majority of the related studies were produced by American authors and institutions in the USA. The keywords frequently used by the authors were physical activity, exercise, barrier, health, male, and adult. In recent years, the most studied trend topics related to physical activity barriers in men were mellitus, health benefits, university students, stigma, glycemic control, time, and facilitator urban. The present study indicated that there is a need for both individual and institutional studies on physical activity barriers in men. The research results showed that there were still significant gaps in understanding physical activity barriers specific to men. Understanding these gaps is crucial for developing effective interventions, creating targeted health promotion strategies, and informing public health policy. This conclusion suggests that while we have a growing body of research on physical activity barriers in men, there is still much research to be conducted in terms of developing a comprehensive understanding and effective interventions to address these barriers.

## 1. Introduction

The COVID-19 pandemic resulted in a spike in scientific research on several topics including inactivity, diseases associated with physical inactivity, both indoor and outdoor physical exercise, environmental aspects, and public health [1,2,3,4]. Restrictions and social isolation measures implemented during the COVID-19 pandemic have made it significantly difficult for men to participate in regular physical activities and have significantly lowered their activity levels [2,5]. Humanity has undergone a significant transformation, which includes wearing masks in public spaces, working in home-based settings, and online education. This transformation has brought the importance of physical activity and inactivity and associated diseases back to the agenda.

Physical activity is recognized as one of the cornerstones of a healthy lifestyle. The World Health Organisation (WHO) recommends that adults should do at least 150 min of moderate exercise or 75 min of vigorous exercise on a weekly basis. However, many individuals face various barriers to performing physical activity [6,7]. Singh et al. [8] in their study addressed the barriers faced by men in physical activity participation. The first step in solving a problem is to diagnose the problem. Therefore, in order to increase men’s participation in physical activities, physical activity barriers should first be identified.

A bibliometric study examining physical activity barriers among men is valuable from many perspectives. Bibliometric studies involve the quantitative analysis of the scientific literature in a particular subject area and thereby contribute by offering information such as research trends, the most-studied topics, leading researchers and institutions, and relationships between publications. This study aims to provide a general mapping of the studies on physical activity barriers in men and to reveal the breadth and depth of scientific research in this field. Furthermore, this study helps to identify research gaps and guide future research by indicating which topics related to physical activity in men have been investigated more and which topics have not been investigated sufficiently. Bibliometric studies provide valuable resources for other researchers and policy-makers by showing which studies and researchers are effective in the field. The gathered data can lay the groundwork for establishing health policies regarding physical activity barriers in men and developing programs that encourage them to be more physically active. Therefore, a bibliometric study provides an important resource for both the scientific community and health policy makers to assess the current state of knowledge on physical activity barriers in men and establish future strategies. Accordingly, the research questions of this study are as follows:

➢ What is the distribution of publications on physical activity barriers in men over the years?
➢ Who are the most influential authors and institutions?
➢ Which countries cooperate more in this field?
➢ What are the most used keywords and trending topics?

## 2. Methods

This study adhered to the Preferred Reporting Items for Systematic Reviews and Meta-Analysis (PRISMA) guidelines [9]. A bibliometric analysis was performed on articles on physical activity barriers in men from January 1992 to May 2024 retrieved from the Web of Science (WoS) database. 

### 2.1. Selection Strategy and Criteria

**Figure 1:**
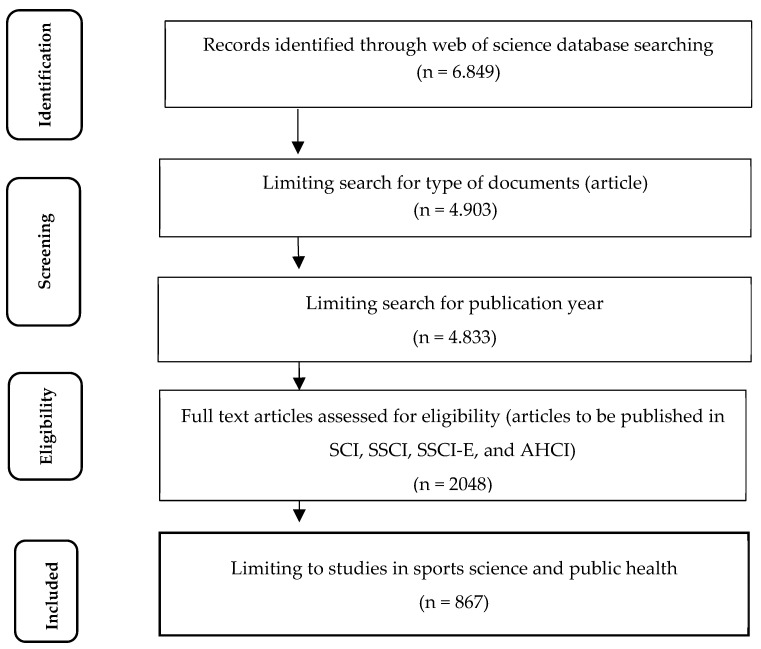
PRISMA flow diagram for identification and screening of the included articles.

The criterion sampling method, which is considered a purposive sampling method, was used to identify the sample of this study. The study included articles that focused on physical activity barriers among men in the Web of Science database and published in SCI, SSCI, SSCI-E, and AHCI. Therefore, as exclusion criteria, the publication types “book note, editorial material, proceedings paper, letter, and correction” were excluded from the study. Consequently, the analysis was completed with 867 articles (Figure 1).

This study, including a bibliometric analysis of 867 studies included in the Web of Science Core Collection database and independently derived by researchers on “Physical activity barriers in men”, was performed by reviewing documents within the framework of descriptive analysis from qualitative research methods. The study addressed both descriptive bibliometrics, aiming to assess productivity, and evaluative bibliometrics, focusing on the assessment of the use of related literature. Descriptive bibliometrics allows one to reveal the distribution and trends of the literature by country, author, year of publication, topic, and language, whereas evaluative bibliometrics allows one to analyze the correlations between authors, publications, and countries through the citations made by authors [10].

### 2.2. Data Analysis Technique

This study aimed to reveal the correlational networks specific to the “physical activity barriers in men” through bibliometric analyses based on the social network analysis method, which allows the identification of authority sources and people in a particular scientific field and the determination of the correlations between them [11,12] and the illustration of these connections [13,14]. This analysis makes it possible to identify the key actors specific to the area of focus, test this structure, and determine the dominant factors influencing its development. In this study, bibliometrix—one of the software tools that can be used to analyze many social networks—was used [15]. The articles that met the inclusion criteria and were included in the study were transferred to bibliometrix via R, a widely used program compatible with the Biblioshiny 4.1 software [16]. This software allowed us to view the literature on physical activity barriers in men. In this study, the Web of Science was used as the database. Access to major international data sources such as the Web of Science, “Science Citation Index (SCI)”, “Social Science Citation Index (SSCI)”, and “Arts & Humanities Citation Index (A&HCI)” provides a significant contribution to bibliometric studies. The Web of Science contains highly important information for bibliometric studies such as the publication year, the publication type, the publication language, the names of authors, the countries where the authors are from, how many references they cite, the abstracts, the keywords, and the bibliographies.

The bibliometric analysis was examined under two main headings during data collection. The bibliometric analysis of physical activity barriers in men was examined under the main topics of general structure analysis and intellectual structure analysis. The diagram below shows the sub-headings focused on data analysis and presents the findings.

#### 2.2.1. General Structure Analysis

This section analyzes articles on physical activity barriers in men according to relevant bibliometric indicators: average number of citations per article, number of authors per article, number of authors and their appearance, co-authors per article, and the authors’ collaboration index. Moreover, the annual publication productivity of the authors and the citation averages, which are related to the number of citations in the studies according to the years of publication, were calculated.

In order to identify the most effective sources in the source analysis, the number of article publications, h-indexes, number of citations, total number of citations (TC), and number of publications (NP) of the journals were analyzed. Another method used in the analysis of academic sources is Bradford’s Law. In this law, journals that contain studies on the subject are divided into three core groups. The journals in the small core group are few and contain important related publications [17]. This core group can be characterized as the most effective journal group in the designated discipline. The second one-third slice contains approximately “n” times as many journals as the core group, and the last slice contains approximately “n2” times as many journals as the core group. The journals in the second group can be considered less effective journals, and the journals in the third group can be considered ineffective journals. In this study, the effective journals were also identified based on Bradford’s law [18], which allows for a systematic analysis of the distribution of literature on physical activity barriers and the identification of the most influential journals in the field. This law is used as an important tool to understand the structure of scientific communication in the field of research and determine future research strategies.

Author analysis took into account the number of related publications of the authors and their h-index values in this area. Also, scientific productivity was calculated based on Lotka’s law, which enables us to identify how many publications the authors who publish in the context of a certain topic have contributed to and, thus, provides researchers with a quantitative estimate of how many more publications can be made in the related literature [19].

In this study, Lotka’s law was used to analyze scientific productivity and author contributions on physical activity barriers in men. This analysis enabled us to reveal research gaps in the field of physical activity barriers and identify future research needs.

#### 2.2.2. Intellectual Structure Analysis

This stage of the study was addressed under three separate headings: conceptual structure, social structure, and intellectual structure.

#### 2.2.3. Conceptual Structure

Co-word analysis is based on the frequency of appearance of keywords of interest in the whole dataset. Its purpose is to map the conceptual structure of a topic using common keywords in a bibliographic dataset. The use of keywords has many advantages, such as covering a wide knowledge base, being objective, and having the opportunity to be more comparative and descriptive in scientific studies [20]. In this study, the common word network was mapped by analyzing the frequency of co-occurrence of keywords together [21], and the areas of interest were examined concerning physical activity barriers in men using clustering techniques. Furthermore, network density and centrality were calculated in the bibliometric analysis of the conceptual structure using keywords. Network density (Q modularity) represents the correlational density and collaboration frequency in each node in the graph [22]. Network density can have a value of 0 to 1. Values closer to 1 indicate closer correlations and connections across clusters. Generally, a Q modularity value between 0.4 and 0.8 is considered an indicator of a good cluster. The degree of centrality signifies the importance of the node’s location in the network and is used to highlight potential key points. A high centrality value means that a node exerts a higher degree of control over other key factors [22].

#### 2.2.4. Social Network Analysis

Social network analysis, also known as network mapping, is a method used to examine network centralization by analyzing nodes and links [23]. In a scientific collaborative network, articles, sources, authors, institutions, or countries represent nodes and co-authors represent links. This is also one of the best-documented forms of scientific collaboration [24]. In order to avoid isolated and “one-off” collaborations in the collaborative network between authors, institutions, or countries, the Louvain method was adopted as the clustering algorithm, the number of nodes was set to 50, and the minimum edge strength was set to 2. Isolated nodes were removed. Finally, collaboration world map analysis was used to demonstrate the social structure. This analysis mainly aims to observe the collaboration and networks across countries. Countries with darker shades publish more than countries with lighter shades. Countries shaded grey indicate that no studies have been produced in that country. The lines indicate the collaboration across countries [25].

#### 2.2.5. Intellectual Structure

Co-citation analysis: In every scientific discipline, some publications assume seminal roles in the development of the related area. These publications, due to their impacts, act as key actors that accelerate such development [26]. Co-citation analysis is utilized to achieve significant features of the research system on which it relies and identify the effects of bibliometric units such as scholars and journals. Co-citation refers to citing two articles together when both articles are cited in a third article. Therefore, co-citation is regarded as the equivalent of bibliographic aggregation. Co-citation analysis helps to follow research trends, identify current issues, and lay the groundwork for future research. Therefore, it is possible to identify effective references, distinguished authors, key institutions, high-ranking journals, and current topics in a field by analyzing co-citation networks [20]. Co-citation analysis has three types: publication co-citation analysis, author co-citation analysis, and journal co-citation analysis [27]. In this study, a co-citation network (sociogram), a graphical representation of the most important and dense correlations among the members of a network represented by “vertices” or “nodes”, was constructed. Vertices are high-frequency items (e.g., cited articles, cited sources, or cited authors) and their size indicates the frequency of their appearance. The smaller the size of a node, the lower the frequency. On the other hand, “lines” or “edges” represent the linking correlation or interaction between vertices in the same article, and their thickness reflects the degree or intensity of co-citation between vertices. The thicker the edges between two vertices, the stronger the connection [22]. While performing the co-citation analysis in this study, the Louvain method was used as the clustering algorithm. The number of nodes was 50 and the minimum edge strength was 20 in the co-citation network for articles, authors, and sources. The historical citation map, which represents the chronological network map in the intellectual structure analysis, was also included in the study.

## 3. Results

### 3.1. Analysis Findings on Descriptive Bibliometrics/General Structure of Data

Basic Information: This section included general statistics on the topic under the headings of Dataset, Source Analysis, and Author Analysis, as well as the year-based distribution of 867 studies on “physical activity barriers in men” in the WOS database, which were obtained independently by two researchers. This section also addressed their collaboration and effectiveness. The reasons for the level of collaboration are the interdisciplinary nature and research complexity. Collaboration covers subjects that require expertise such as physical activity barriers, physiology, psychology, sociology, and public health and different methodological approaches. It needs large sample groups and includes studies that require long-term follow-up.

In this study, bibliometric analysis was carried out with 867 articles on physical activity barriers in men between 1992 and 2004. These articles showed an average of 27.67 citations per article between 1992 and 2004. They were written by 4242 authors, with an average of 0.204 articles per author from 397 sources. Twenty-four of these authors published their single-authored studies under a single title. From 1992 to 2024, 867 articles on physical activity barriers in men were cited 31,597 times by studies reviewed on WOS. This situation shows that the subject is a current and developing research area, and multi-authored studies were dominant. The abundance of one-off studies indicates the scarcity of sustainable research programs. Based on these data, it is shown that the subject area is still in its development phase, and more specialized researchers are needed. It also points to the necessity of sustainable research programs and the importance of continuing international collaborations. Therefore, these data show that the field is developing but that more systematic research is needed.

**Figure 2:**
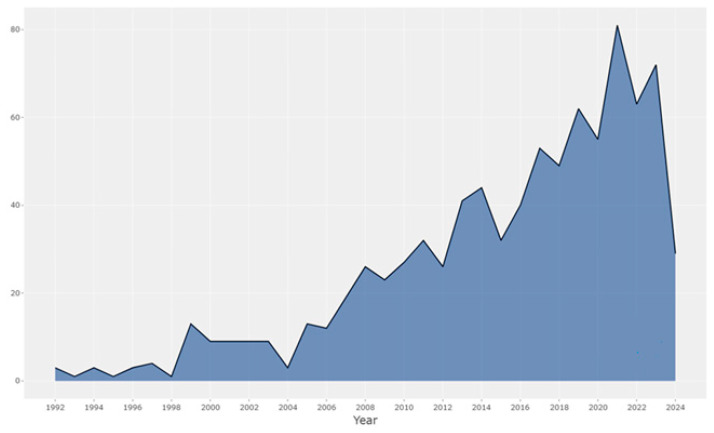
Articles.

The reasons for the increase in rates of publications regarding physical activity barriers after 2020 include the increase in health concerns arising from the effect of the COVID-19 pandemic. The COVID-19 pandemic caused the closure of sports facilities and the extension of the stay-at-home period, along with curfews and quarantines. This was associated with changes such as the transition to an online working order as well as the increase in awareness of male health problems, the increase in obesity rates, and the increasing interest in the effects of physical activity on the chronic immune system (Figure 2).

Figure 2 shows the graph of publication rates of scientific studies specific to the physical activity barriers in men in the years between 1992 and 2024.

It was observed that the publication rate of articles on physical activity barriers in men rapidly increased, especially after 2004, and more than half of the total number of articles were published after 2012. The number of studies conducted after 2020 increased, and this finding may suggest that physical activity barriers are one of the trending topics studied recently. 

**Figure 3:**
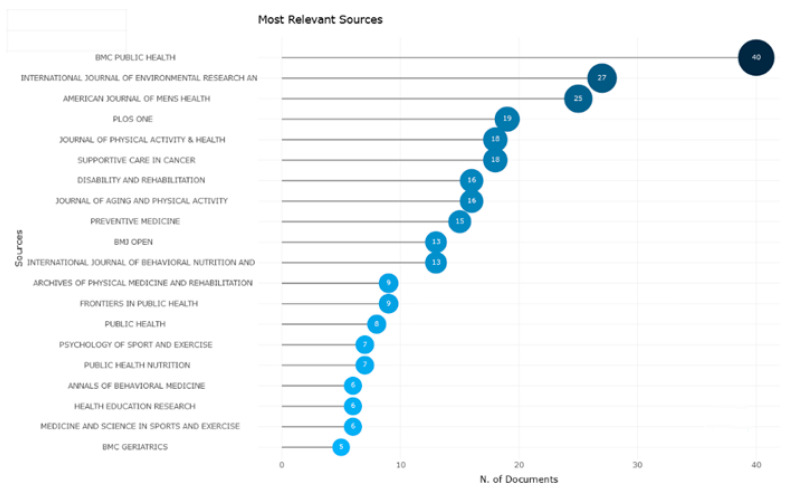
Most relevant sources.

Figure 3 shows the most relevant journals on physical activity barriers in men.

The most productive journal for article publication was “BMC Public Health”, with 40 articles between 1992 and 2024. This journal was followed by the “International Journal of Environmental Research and Public Health”, with 27 articles, and the “American Journal of Men’s Health”, with 25 articles.

**Figure 4:**
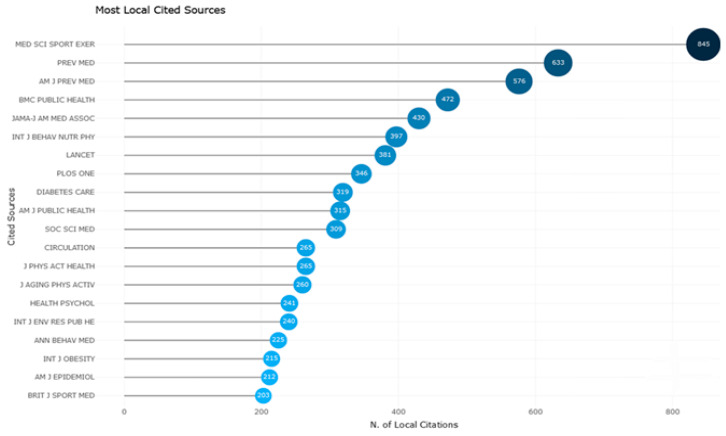
Most cited sources (from the reference lists).

The most cited journal in the references of all articles included in the study was ‘Medicine and Science in Sports and Exercise’ with 845 citations, followed by “Preventive Medicine” with 633 citations, and the ‘American Journal of Preventive Medicine’ with 576 citations. The graphic based on Bradford’s law showing the distribution of the literature on physical activity barriers in men among the journals is presented below (Figure 4).

**Figure 5:**
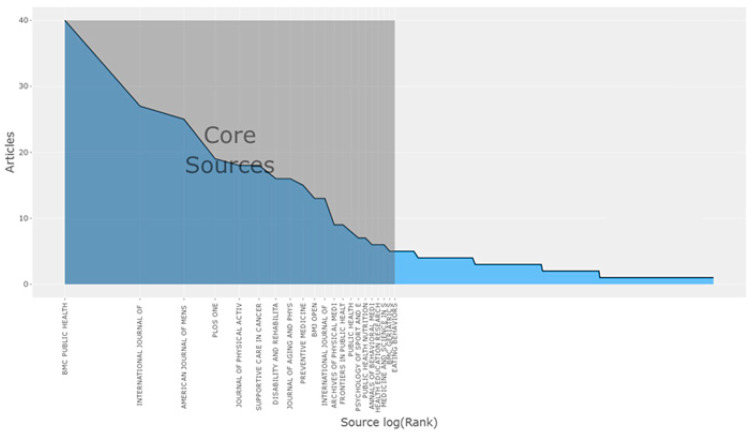
Bradford’s law.

According to Bradford’s Law, the areas covered by the BMC Public Health journal in basic sources were more than the other journals. In other words, it was included in the core journals. This journal was followed by the International Journal of Environmental Research and Public Health and the American Journal of Men. These journals were similar to the list of journals with the highest number of publications (Figure 5).

**Figure 6:**
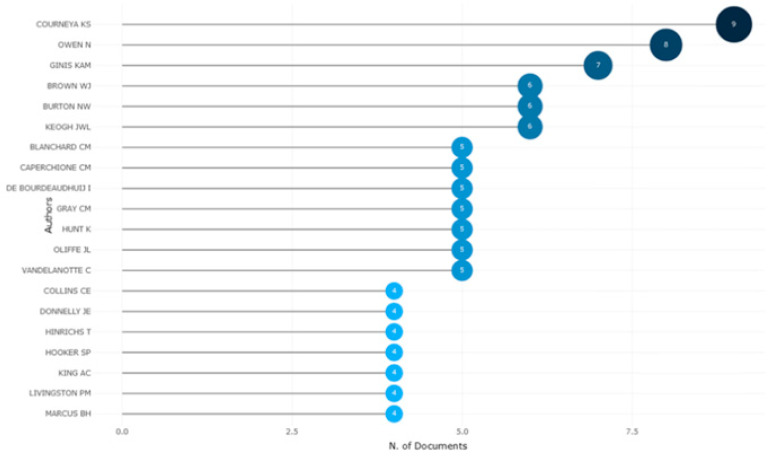
H-index values of the authors.

In the analyses related to the authors who conducted studies on physical activity barriers in men, their h-index factors were assessed. Figure 6 shows the graph related to these findings.

**Figure 7:**
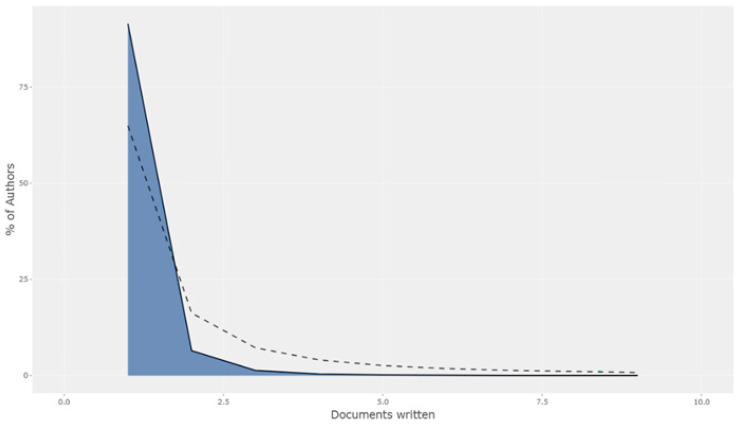
Distribution of scientific productivity according to Lotka’s law.

The H-index shows how many ‘h’ publications of an author have been cited for at least ‘h’ number of times and indicates not only the number of citations but also the consistency of these citations [28]. Courneya K. S. was the author with the highest h-index (h-index = 9) among the authors who published articles on physical activity barriers in men. Courneya was followed by Owen N. (h-index = 8) and Ginis K. A. M. (h-index = 7). The similar h-index values of the three authors with the highest number of publications proved that all these authors were key actors in this subject. Figure 7 shows the graph drawn based on Lotka’s law regarding the scientific productivity of the authors.

The findings of this study showed that 93% of the researchers who published articles on physical activity barriers in men published only one article, and the rate of authors who published two articles was 5%. Moreover, 2% of the researchers published three or more studies on this subject. Lotka’s law states that the ratio of researchers contributing to a subject area with a single publication to all publications should be 60%. The ratio of researchers who contribute with two publications to those who contribute with a single publication should be one-quarter, and the ratio of researchers who contribute with three publications should be one-ninth [29]. Accordingly, the articles published on physical activity barriers in men in WOS violate Lotka’s law. Authors generally preferred to conduct research on physical activity barriers once, and the number of authors who specialized in that topic remained limited. In this sense, when the related studies were analyzed according to Lotka’s law, it was concluded that the related literature should be improved (Figure 7).

### 3.2. Conceptual Structure

**Figure 8:**
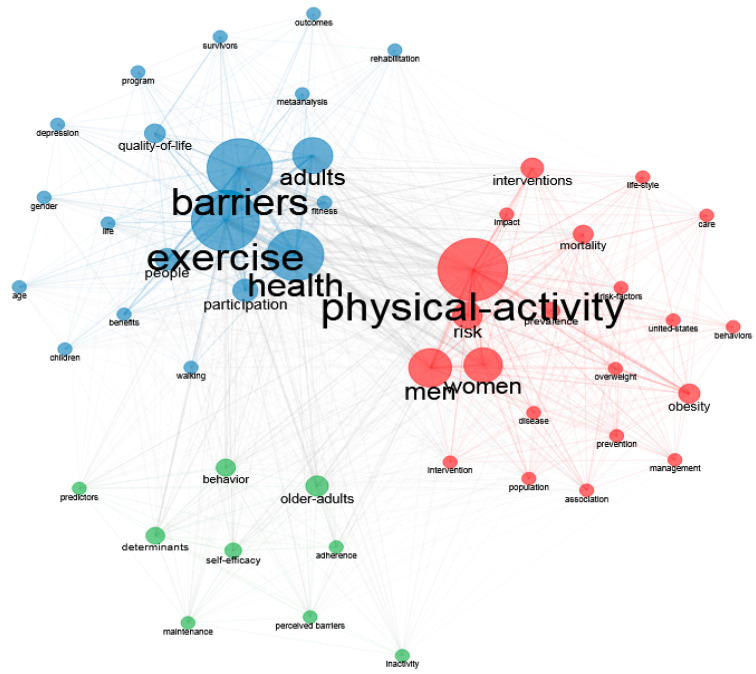
Keywords.

Through common word analysis, the most frequently used concepts were identified in the keywords of 867 studies published on “physical activity barriers in men”. These keywords provide researchers with clues about the most frequently studied topics in the research area and the changes in these topics over the years. Moreover, researchers can also find clues about current trends using this analysis. Figure 8 shows the correlations and formation networks of the 50 most frequently used keywords. Each ellipse represents a keyword in the image created using the Louvain Clustering Algorithm, while the thickness of the lines between the ellipses varies directly proportional to the intensity of the correlation between the words. Figure 8 shows the major words used over time regarding physical activity barriers in men. This analysis demonstrated the presence of four different clusters of keywords used in our study, where these terms are based on the same topics (co-occurrence). The keyword with the highest centrality and density was ‘Physical activity’.

In Figure 8, the size of the circle is proportional to the total frequency of the KWP included in that cluster. In this sense, when the sizes of the circles in Figure 8 were analyzed, “Physical activity” was found to be the most recurrent keyword.

**Figure 9:**
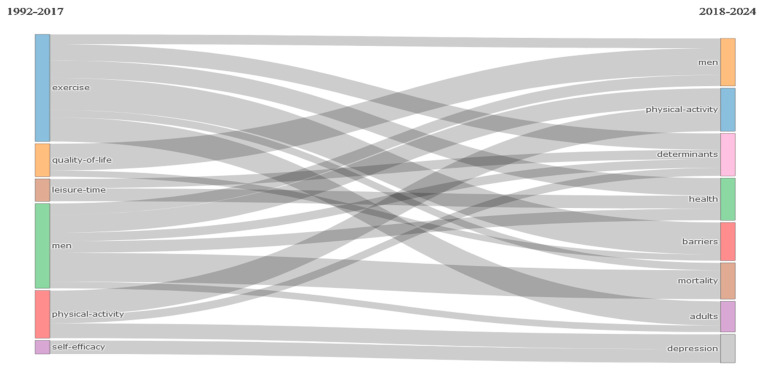
Time periods.

According to the time periods set out in the figure above, exercise, the most popular keyword between 1992 and 2017, evolved toward men, physical activity, determinants, and health, while maintaining its popularity (Figure 9).

**Figure 10:**
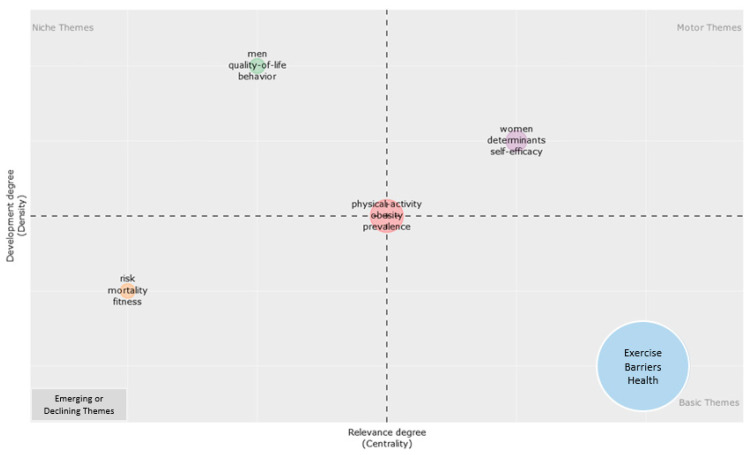
Development degree.

Figure 10 represents the centrality (i.e., the degree of interaction of a network cluster compared to the other clusters) of the X-axis and provides information about the importance of a theme. The y-axis symbolizes density (i.e., measures the internal strength of a cluster network and can be considered as a measure of theme development). According to Figure 10, it was found that the themes in the upper-right quadrant (women’s determinants of self-efficacy) showed development on physical activity barriers and were important for constructing the research area. These themes were the motor themes of the subject area of physical activity barriers, had high internal consistency, and were conceptually closely correlated with each other. In the lower-right quadrant, it was identified that the important and basic themes for physical activity barriers were correlated to health. In the upper-left quadrant, the marginal themes were identified as men, quality of life, and behavior, while the themes just below (lower-left quadrant), which are known to be both poorly developed and marginal, were identified as risk, mortality, and fitness.

**Figure 11:**
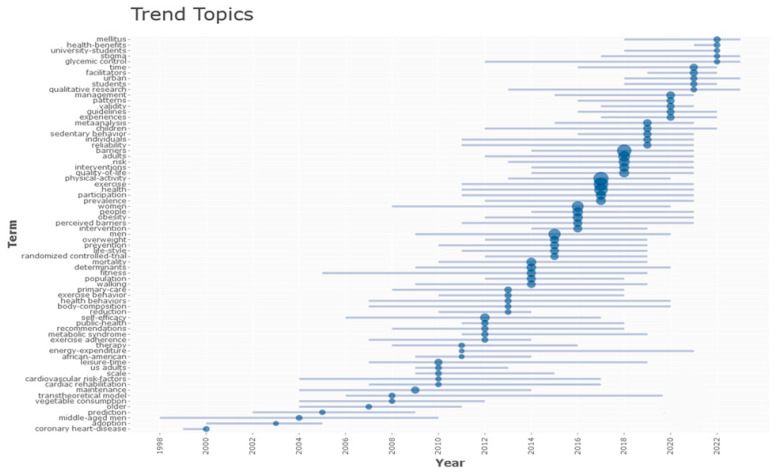
Trending topics featured by year.

Figure 11 illustrates the concepts that appeared as trending topics in the studies published in the subject area of measurement invariance over the last 10 years.

When Figure 11 was analyzed, it was observed that the studies on the physical activity barriers in men after 2020 were mainly concentrated on the trending topics associated with mellitus, health benefits, university students, stigma, and glycemic control.

### 3.3. Social Structure

**Figure 12:**
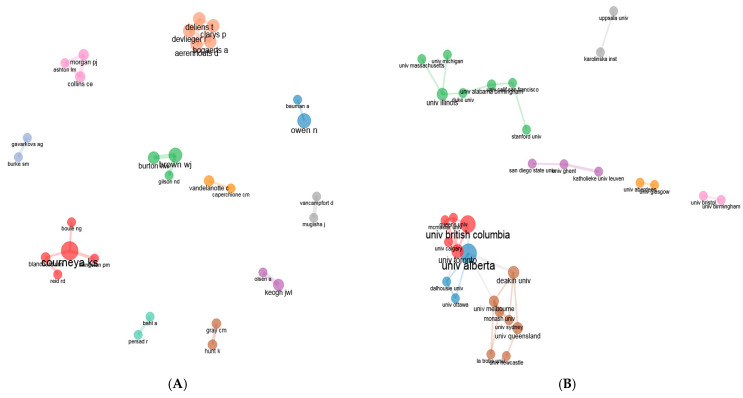
(**A**) The authors and (**B**) their institutions.

In the bibliometric mapping study, the nodes in the network show the authors, institutions, and countries, as well as the links between them show their collaborations. The size and thickness of the circle around the nodes in the network in the images indicate the degree of centrality of that actor in the network. In the social structure of 867 articles, there was one different cluster of authors (Figure 12). “Courneya” was the most prolific author. On the other hand, seven different clusters of institutions were established, with “University British Columbia” in cluster 1, “University Alberta” in cluster 2, and “University Illinois” in cluster 3.

**Figure 13:**
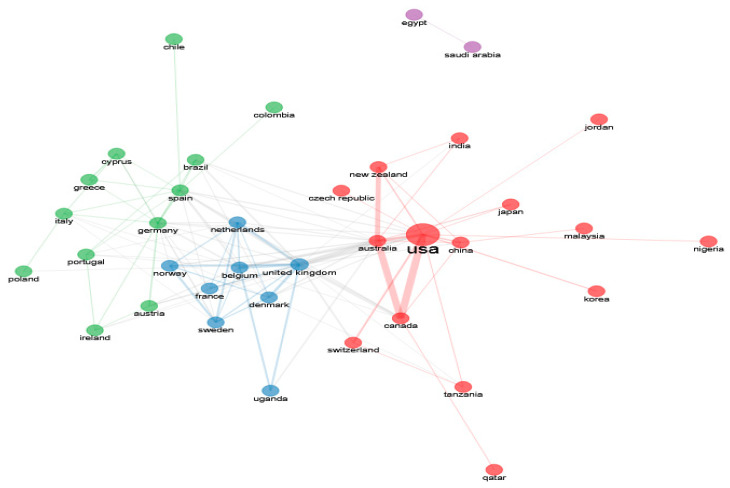
Countries.

Figure 13 shows that the United States of America had the highest centrality in the collaboration network between countries. Therefore, it could be stated that the United States of America serves as the headquarters for university-based scientific research on physical activity barriers in men.

**Figure 14:**
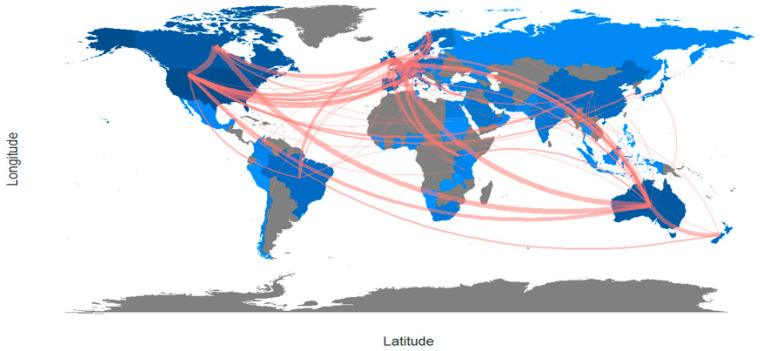
International collaboration.

The international collaboration of countries in the research field was quite intense (Figure 14). Many studies have been conducted with international collaboration on physical activity barriers in men. The USA and Canada had the highest number of international collaborations. The links between these countries and other countries are also noticeable on the map. The top three collaborations among the countries in the dataset of 867 articles concerned the United States–Canada, Canada–Australia, and England–Canada.

## 4. Discussion

In this study, bibliometric analysis, as a quantitative analysis method, was used to map, illustrate, and interpret the data by demonstrating the structure and development of research on physical activity barriers in men. Both types of bibliometric methods are preferred due to their ability to analyze the topic in depth and demonstrate the development of the topic in the subject area. In this study, studies in the WOS database were taken into consideration. The phrase “physical activity barriers in men” was entered into the database, and 867 articles published between 1992 and 2024 were organized for analysis. The findings of the study were analyzed using the Biblioshiny interface through the R programming language.

Moreover, 867 articles in 397 journals on physical activity barriers in men published between 1992 and 2024 were produced by 4242 authors. The average number of articles produced annually on physical activity barriers in men was 8.59. Although the annual citation rate was 2.36, the average number of citations per article was 27.67. Twenty-four of these articles were written by a single author. Accordingly, it could be asserted that the field was dominated by studies with two or more authors on physical activity barriers in men.

The 27.67 citations per article may indicate that the research has a certain impact.

The fact that the most-cited study received 642 citations indicates that some basic research is important for the field. Although the annual average of 2.363 citations may be considered low, the concentration of citations in certain studies may indicate a lack of long-term interest. Although publications have been conducted on the subject since 1992, a spike occurred after 2004, indicating that the field is still in the development stage and basic research is gradually reaching a better level. When the number of articles by year was analyzed, it was observed that the number of articles followed an upward trend. The annual citation rates of the articles followed a fluctuating trend. It was concluded that studies on the topic should be improved both qualitatively and quantitatively.

Examining the ranking of journals on physical activity barriers in men, it was observed that the most productive journal was ‘‘BMC Public Health’’ with 40 articles and was among the main sources. The most prolific author was Courneya K. S., with a total of nine articles. The most cited study was “Environmental and policy determinants of physical activity in the United States” published by Brownson et al., with 642 citations. It was found that the articles published in WOS on physical activity barriers in men were not in accordance with ‘Lotka’s law’. The authors preferred to conduct one study on physical activity barriers in men, and the number of authors who specialized in the topic remained limited. This is caused by methodological and structural reasons. Methodological reasons include the multidisciplinary nature of the field of study, temporary participation of researchers from different fields of expertise, and one-time studies resulting from difficulties in data collection and analysis. For structural reasons, the immaturity of the field, lack of long-term research programs, discontinuity of funding sources, and inadequacy of institutional support come to the forefront.

In the WOS database, the most commonly used keywords in studies on physical activity barriers in men were physical activity, exercise, barriers, health, and man, respectively. The trending topics associated with the topic in 2022 were mellitus, health benefits, university students, stigma, and glycemic control. When the graph of trending topics related to physical activity barriers in men was analyzed, the concept of fitness took first place in trending topics from 2004 to 2020 but reached the peak level in 2004. While barriers, time, facilities, and management topics related to physical activity barriers were trending between 2014 and 2020, it was observed that health-related topics were trending after 2020.

The analyses based on countries indicated that the USA conducted the most studies on physical activity barriers in men. Hence, given the intensive collaborative network among other countries in the intellectual structure, it could be asserted that the USA is the main actor. Furthermore, the University of British Columbia (n = 42) was the institution that conducted the highest number of studies on the topic. Based on this finding, a great majority of the studies on physical activity barriers in men were produced by American and Canadian authors and institutions in these countries.

Moreover, the international collaboration of countries in the research field was quite intense, indicating how much the topic of physical activity barriers in men is worth studying in the international literature. However, some regions were underrepresented in this area. This may be due to a lack of research infrastructure. Countries that lack sufficient resources in areas such as public health or sports science may not prioritize such research. On the other hand, in some regions, a focus on physical activity or men’s health may lack cultural emphasis or encounter social cohesion. This may lead to a decrease in research output. Furthermore, English-dominant scientific journals may limit participation from non-English-speaking countries. International partnerships may be an outcome of a study that addresses cultural, ethnic, and racial differences among societies. Actions such as increasing international cooperation, providing special funds for underrepresented countries, and providing opportunities to broadcast in different languages can be taken to fill these gaps.

This study has some limitations. The study was conducted by analyzing data collected from only one specific database, the WOS database. It is recommended that this study be compared with searches conducted in other preferred databases. Furthermore, the R programming language was used in this study. Different analyses can be performed with different programs, such as VOSviewer and Cite Space.

## 5. Conclusions

This study evaluated articles related to physical activity barriers in men in the field of health and sports sciences. The first step in solving a problem is to identify it correctly. Therefore, it is recommended to conduct new studies on physical activity barriers in men at the individual and organizational levels to provide important evidence for preventing inactivity and the spread of diseases related to inactivity and support them with quantitative–qualitative or mixed analyses. The studies generally focused on young and middle-aged men, and the number of studies on older men is limited. Factors such as mobility limitations due to aging, chronic diseases, and social isolation may be important barriers in this population. Future studies could focus on strategies to increase physical activity participation in older men. Psychological factors such as a lack of motivation, depression, and stress may also be important for men regarding their participation in physical activity. These areas deserve further investigation. Furthermore, information is needed on barriers to physical activity in men from different cultural and ethnic groups. The impact of social and cultural factors on physical activity could be investigated, particularly in immigrant, minority, and indigenous communities.

The study only evaluated articles on physical activity barriers in men in health and sports sciences. Future studies should be more comprehensive and include other publication types (announcements, books, book chapters, and research projects). The results of the study showed that there is a need for both individual and institutional studies on physical activity barriers in men. It is recommended that new studies focus on preventing inactivity, understanding diseases related to inactivity, and addressing the research problem in this direction. There are still significant gaps in understanding physical activity barriers specific to men. Understanding these gaps is extremely important for developing effective interventions, creating targeted health promotion strategies, and informing public health policy. While we have a growing body of research on physical activity barriers in men, there is still much work to be conducted in terms of developing a comprehensive understanding and effective interventions to address these barriers.

## Data Availability

The data presented in this study are available on request from the corresponding author.
